# Comparative Study of MgO Nanopowders Prepared by Different Chemical Methods

**DOI:** 10.3390/gels9080624

**Published:** 2023-08-02

**Authors:** Ligia Todan, Luminița Predoană, Gabriela Petcu, Silviu Preda, Daniela Cristina Culiță, Adriana Băran, Roxana-Doina Trușcă, Vasile-Adrian Surdu, Bogdan Ștefan Vasile, Adelina-Carmen Ianculescu

**Affiliations:** 1 Institute of Physical Chemistry “Ilie Murgulescu” of the Romanian Academy, 202 Splaiul Independenței, 060021 Bucharest, Romaniaadibaran@gmail.com (A.B.); 2 Department of Science and Engineering of Oxide Materials and Nanomaterials, Faculty of Chemical Engineering and Biotechnologies, “Politehnica” University of Bucharest, 1-7 Gh. Polizu, 011061 Bucharest, Romaniaadrian.surdu@upb.ro (V.-A.S.); bogdan.vasile@upb.ro (B.Ș.V.);

**Keywords:** MgO nanopowders, sol–gel method, microwave-assisted sol–gel method, hydrothermal method, photocatalytic degradation of methyl orange dye

## Abstract

Magnesium oxide (MgO) was synthesized by three different methods: the sol–gel (SG), microwave-assisted sol–gel (MW), and hydrothermal (HT) methods for comparing the influence of the preparation conditions on the properties of the products. The powders were annealed at 450 °C. The samples were characterized by X-ray diffraction, scanning electron microscopy (SEM), transmission electron microscopy (TEM/HRTEM), selected area electron diffraction (SAED), energy-dispersive X-ray spectroscopy (EDX), BET specific surface area and porosity, photoluminescence, and UV–Vis spectroscopy. The samples consisted mainly of periclase as a crystalline phase, and the MW and HT preparation methods generated particles with higher specific surface areas. The powders had less-defined morphologies and high levels of aggregation. The optical band gaps of the samples were determined from UV DRS, and the photocatalytic activities of the magnesium oxides obtained by the three methods towards the degradation of methyl orange (MO) under UV light irradiation was evaluated.

## 1. Introduction

New and emerging fields of technology have provided materials prepared in the form of small particles with remarkable properties, such as being photocatalytic in nature, possessing a high surface area, being electrostatic, having a tunable pore volume, being magnetic, being hydrophobic and hydrophilic, etc., with novel potential applications. A high surface-to-volume ratio, which is characteristic of nano- and micro-particles, controls the interaction with pollutants and bacteria, conferring an increased efficiency compared to bulk materials [1,2].

Nanostructured metal oxides have a wide range of applications, including for catalysis and electronic and photonic devices [1,3,4]. They show exceptional potential for the photocatalytic breakdown of pollutants as they are considered safe, insoluble in water, and biologically inert [5,6,7]. Magnesium oxide (MgO), a wide band gap insulator, has been used primarily as a ceramic material in thermal engineering and heating elements and for refractory purposes. Fundamental and application studies have developed less conventional ways of exploiting its properties, which include catalysis, in toxic wastes remediation, and as an antibacterial agent. Micro- and nano-structured oxide powders have different properties from the bulk material form, and their reactivity capability is due to surface defects that include low coordination ions and/or vacancies [1,3]. The various defects created in magnesium oxide’s structure are considered intermediate energy levels inside the band gaps. In principle, a decrease in band gap energy suggests the possibility that photogenerated electrons from the conduction band of MgO are transferred to the defect centers, thus preventing their recombination with the h^+^ species. This effect contributes, along with other properties that can be varied from the synthesis method (porosity, surface area, morphology, and size), to the high photocatalytic activity of magnesium oxide, as has been reported in the literature [8,9,10,11,12]. In addition, the ability of MgO nanoparticles to generate reactive oxygen species, such as ·O_2_^−^ and ·OH, confers them antibacterial properties [9,13,14], and their porous nature makes them suitable for waste remediation, allowing the adsorption of pollutants [15,16].

Several preparation methods for magnesium oxide have been developed with the aim of generating nanoscale particles with active surfaces, including dehydration of the Mg(OH)_2_, thermal decomposition of various precursors [17], the sol–gel method [18,19], chemical vapor deposition [20], the hydrothermal method [21], and surfactant methods [22].

Thus, developing a simple procedure for preparing small-sized MgO powders under mild conditions has remained a challenging topic of investigation [1].

The sol–gel process is a bottom-up synthesis method in which the final product is formed by several irreversible reactions. The reaction rate depends on various factors such as pH, concentration, type of solvent, and temperature. Sol–gel-generated MgO powders have high surface areas and particular morphological and physical properties [23,24].

Hydrothermal precipitation is another promising method because the material resulting under mild reaction parameters can be nanostructured, with high crystallinity and various morphologies. Samples of MgO prepared using hydrothermal synthesis show emission peaks at 395 nm and 475 nm, and these are due to surface defects since nanoparticles exhibit a quantum confinement effect [25,26].

Of the many methods employed for the fabrication of MgO, microwave irradiation has recently gained interest over conventional methods due to its short duration, small investment, and its success in manipulating the morphology and architecture of nano- and micro-structures [27,28]. The effect of particle size, pH, and form of active MgO species obtained by microwave-assisted synthesis on the oxide’s properties has been demonstrated by showing its bactericidal performance in an aqueous environment due to the superoxide formation [24,29].

A brief literature survey regarding the chemical methods of MgO preparation in solution is presented in Table 1. The hydrothermal and sol–gel methods are well-represented in the literature, with a variety of precursors and synthesis parameters. The microwave irradiation method is less common due to its novelty statism but is increasingly used due to its advantages, as described above.

In our study, for comparison, MgO was obtained by three methods: sol–gel, hydrothermal, in which Mg(NO_3_)_2_ × 6H_2_O was the precursor treated with a precipitating agent, and microwave-assisted sol–gel, which involved Mg(CH_3_COO)_2_ × 4H_2_O precipitation with ammonia. The structure and morphology of the oxide powders obtained by these synthesis methods were characterized. Their photocatalytic activities were evaluated by monitoring the photodegradation of methyl orange dye as a model pollutant under UV light irradiation, and the band gaps were determined. The results were discussed comparatively.

## 2. Results and Discussion

MgO powders prepared by the sol–gel, hydrothermal, and microwave-assisted sol–gel methods were investigated. An attempt was made to compare their structural and morphological properties and correlate them with their photocatalytic activities.

### 2.1. Phase Composition Investigation

**Figure 1:**
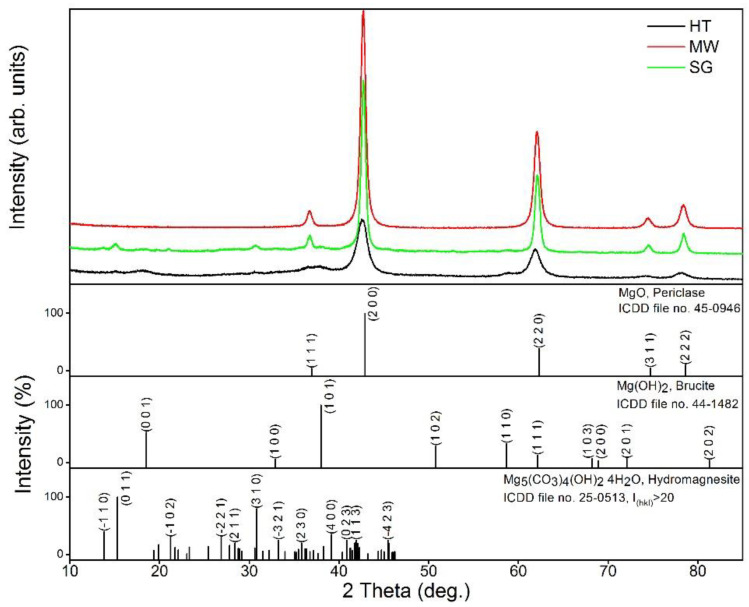
XRD patterns of the samples prepared by the microwave, sol–gel, and hydrothermal methods thermally treated at 450 °C.

Figure 1 presents the XRD diffractograms of the three thermally treated samples at 450 °C, depending on the preparation method. The main crystalline phase in all three samples was periclase, MgO, according to ICDD file no. 45-0946. The sample prepared by the microwave method contained single-phase periclase. The samples prepared by the sol–gel and hydrothermal methods also contained phases of brucite (ICDD file no. 44-1482) and hydromagnesite (ICDD file no. 25-0513) in addition to the main periclase phase. The quantitative ratios between the three phases, determined by the RIR (reference intensity ratio) method, are listed in Table 2.

Table 2 contains information regarding the crystalline phases present in the samples, the lattice parameters (only for periclase), the crystallite sizes (*D*_XRD_) calculated by the Scherrer method, and the quantitative ratios.

The sample prepared by the sol–gel method had the largest crystallite sizes for the periclase phase while the smallest crystallite sizes for the periclase phase were found in the sample prepared by the hydrothermal method. Regarding the lattice parameters of the unit cells of the periclase crystals, the smallest values were determined for the sample prepared by the sol–gel method while the largest values were found in the sample prepared by the hydrothermal method.

### 2.2. Morphological, Structural, and Elemental Analyses

**Figure 2:**
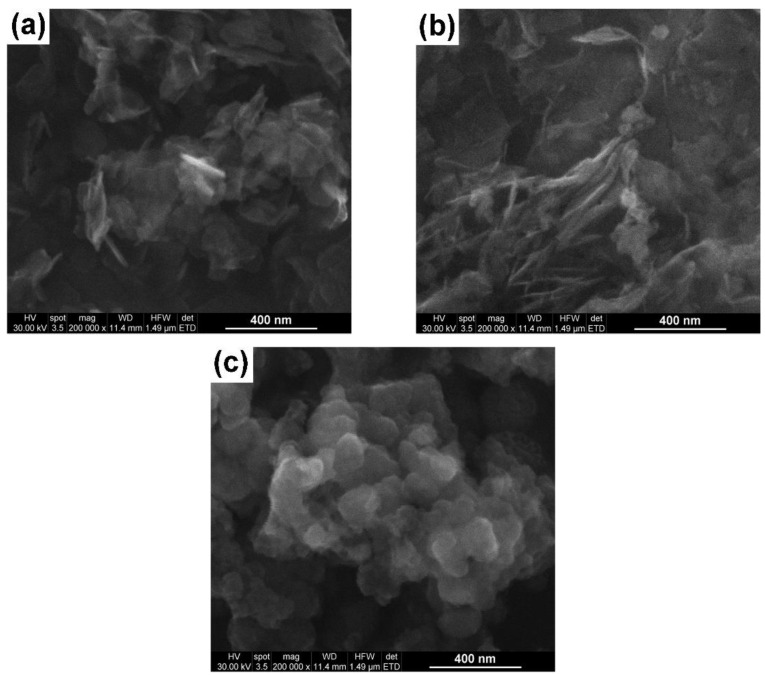
SEM images of the MgO particles: (**a**) MgO HT, (**b**) MgO SG, and (**c**) MgO MW.

Figure 2 shows SEM images of the MgO particles prepared by the three above-mentioned methods. Both the hydrothermally synthesized and the sol–gel MgO powders consisted of non-uniform particles in terms of their sizes, with no clearly defined shapes (Figure 2a,b). Unlike them, the powdered sample synthesized using the microwave procedure exhibited well-defined, nearly spherical MgO particles, with sizes ranging between 80 and 120 nm (Figure 2c). To better observe the details regarding the morphological features and crystallinities of the particles prepared by the three mentioned methods, TEM, HRTEM, and SAED investigations were performed.

**Figure 3:**
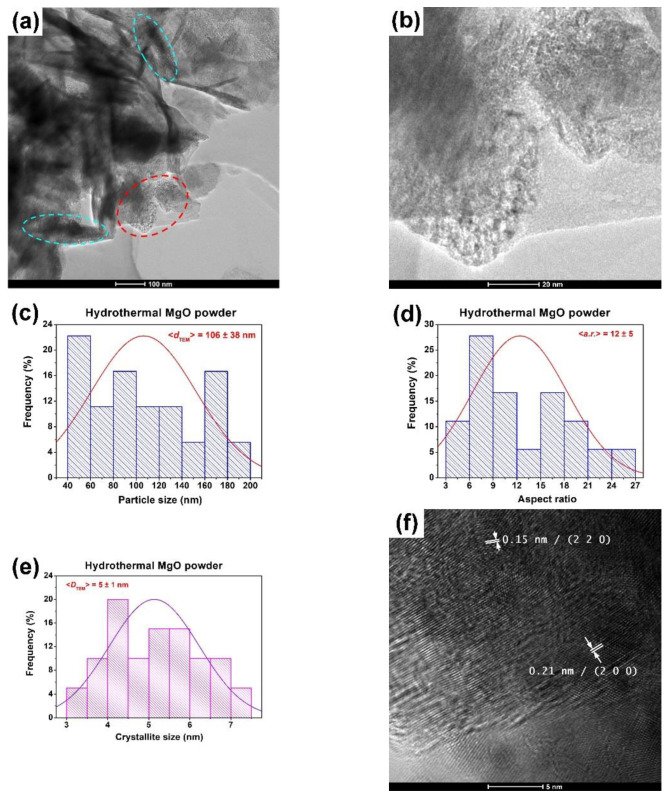

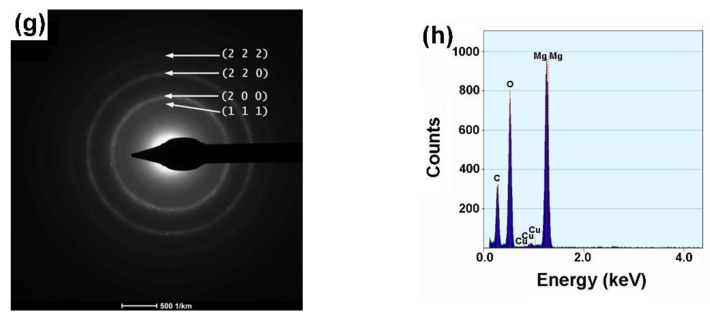
Low (**a**) and high (**b**) magnification TEM images (dashed red ellipse indicates the tabular particles and dashed cyan ellipses rod-like structures); size distribution of the tabular aggregates (**c**); aspect ratio (*a.r.*) distribution of the rod-like aggregates histogram (**d**); crystallite size distribution histogram (**e**); HRTEM image (**f**); SAED pattern (**g**); and EDX spectrum (**h**) of the hydrothermally prepared MgO powder.

The bright–field TEM images showed that there were significant morphological differences depending on the preparation route. The hydrothermally prepared sample consisted of unevenly sized thin MgO particles, most of which had a tabular and rod-like morphology (Figure 3a), with their crystallinities confirmed by the long-range ordered fringes revealed by the HRTEM image shown in Figure 3f. It is worth mentioning that the so-called “tabular particles” actually represented aggregates with an average size (*d*_TEM_) of 106 ± 38 nm, as indicated in the histogram in Figure 3c. These aggregates were formed by a 2D self-assembly process of very small polyhedral crystallites with sizes well below 10 nm, as the enlarged image (Figure 3b) corresponding to the dashed red ellipse in Figure 3a reveals. A value for the average crystallite size in the HRTEM images could be only roughly estimated because of the tendency of the crystallites to overlap, which prevented a clear identification of their outlines. Nevertheless, the estimated values of the crystallite sizes (*D*_TEM_ = 5 ± 1 nm) (Figure 3e) were close (within the error bar) to those determined from the XRD data, which indicated the smallest crystallite size (*D*_XRD_ = 6 nm) for this sample. On the other hand, the rod-like particles seemed to appear when, towards the end of the hydrothermal treatment, the spherical structures collapsed and recrystallized preferentially on the c–face of the crystallites [9]. As with the tabular particles, these rod-like structures of various values of length, L, and width, l, were also aggregates of small crystallites (the dashed cyan ellipses in Figure 3a). An average value of the aspect ratio (expressed as L/l and noted as *a.r.*) of 12 ± 5 was determined from the measurements carried out on ~40 rods from different areas of the TEM images (Figure 3d). A certain amount of amorphous phase was also present in this powder as the continuous and slightly diffuse aspect of the concentric rings in the related SAED image shows (Figure 3g).

**Figure 4:**
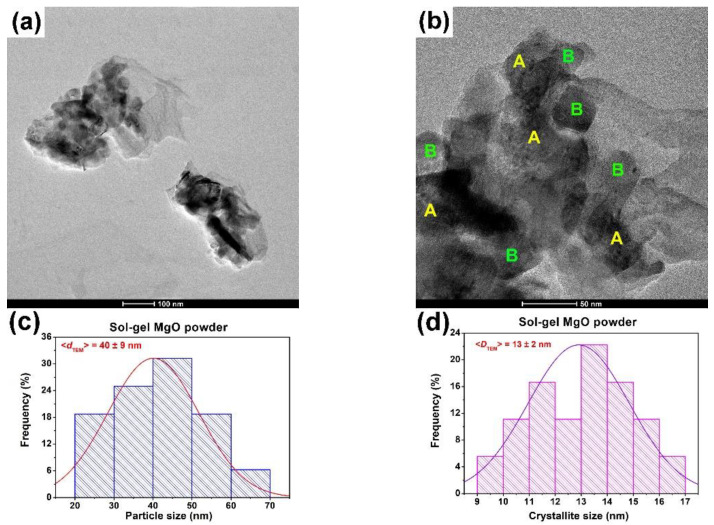

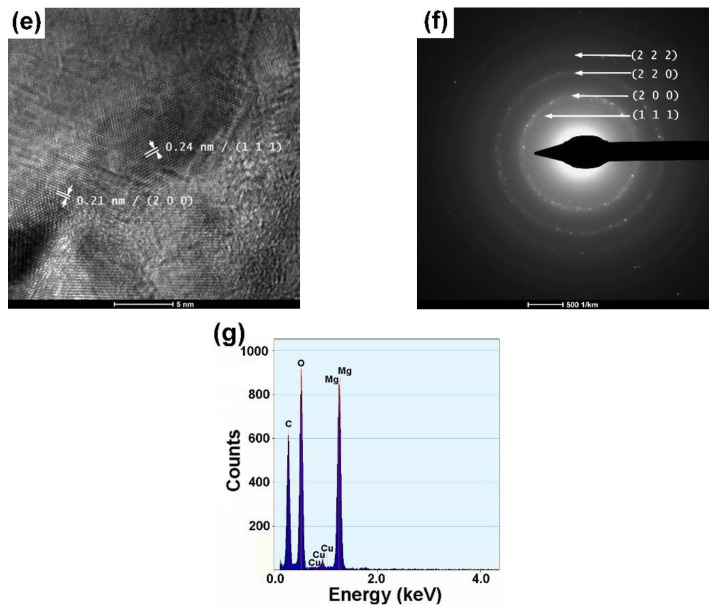
Low (**a**) and high (**b**) magnification TEM images (A corresponds to thin foils and B to individual particles of polyhedral shape); size distribution of the aggregates histogram (**c**); crystallite size distribution histogram (**d**); HRTEM image (**e**); SAED pattern (**f**); and EDX spectrum (**g**) of the sol–gel MgO powder.

The powdered sample prepared by the sol–gel method exhibited a duplex-type morphology (Figure 4a) where elongated or enlarged thin foils (denoted by A), structured as nanosized crystallites, coexisted with individual particles (denoted by B) of polyhedral shapes with well-defined edges and rounded corners. These particles appeared to be single crystals as they showed no structuring, as can be observed in the higher magnification TEM image in Figure 4b. For the individual particles, an average size (*d*_TEM_) of 40 ± 9 nm was estimated (Figure 4c). In this case, the crystallites that built up the thin foils appeared to be slightly larger in size (*D*_TEM_ = 13 ± 2 nm) (see Figure 4d) than those observed for the hydrothermal powder discussed above. The HRTEM image in Figure 4e and the SAED pattern in Figure 4f also reveal an increased crystallinity which showed small bright spots that formed the diffraction rings.

**Figure 5:**
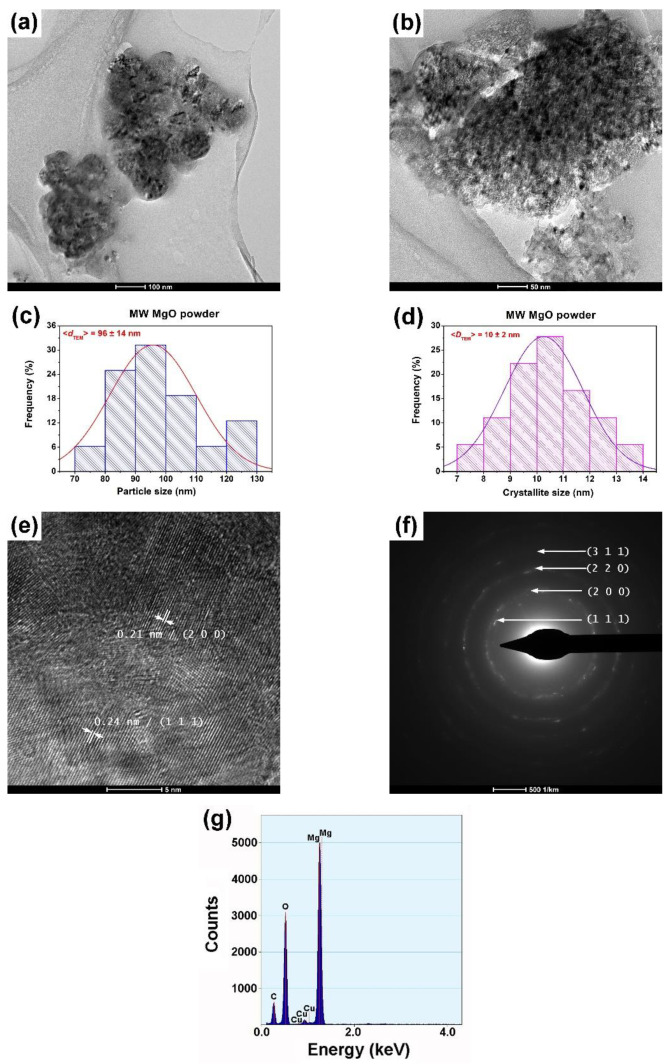
Low (**a**) and high (**b**) magnification TEM images; size distribution of the aggregates histogram (**c**); crystallite size distribution histogram (**d**); HRTEM image (**e**); SAED pattern; (**f**) and EDX spectrum (**g**) of the MW-assisted MgO powder.

By microwave irradiation, the basic magnesium oxide particles, acting as nucleation sites, gradually formed the small and dispersed initial centers that aggregated, generating microspheres [27]. Indeed, the TEM image in Figure 5a shows that the single-phased MgO powder prepared by this technique consisted of nearly spherical aggregates with an average size (*d*_TEM_) of 96 ± 14 nm (Figure 5b). A look inside these aggregates revealed their structuring in small, polyhedral crystallites (Figure 5c), somewhat similar to those observed in the sol–gel powder. An average crystallite size value of *D*_TEM_ = 10 ± 2 nm, similar to that determined from the XRD data (Table 1), was found in this case (Figure 5d). Therefore, based on the TEM investigations, we concluded that the particles from the SEM image in Figure 2c were aggregates of crystallites strongly bound together. In addition, for this powder, the related HRTEM image showed a high crystallinity degree (Figure 5e). However, the more diffuse aspect of the diffraction rings in the corresponding SAED pattern, consisting of fewer bright spots than the sol–gel powder, indicated an intermediate crystallinity (somewhat lower than that of the sol–gel powder but higher than that determined for the hydrothermally processed MgO sample), which was in agreement with the values estimated for the average crystallite size (Figure 5f).

During microwave-assisted combustion synthesis, gaseous products are released that create cavities in the initially formed oxide microspheres, generating defects that could act as catalytic sites [27]. Nano- and micro-scale materials (Figure 2) have large surface-to-volume ratios, resulting in the formation of voids on the surface as well as inside the agglomerated particles that cause absorption bands in the UV regions [25].

The EDX spectra of all three MgO powders under investigation showed the high chemical purity degrees of the samples (Figure 3h, (Figure 4g and (Figure 5g). Apart from the carbon and copper atoms related to the TEM grids, only the presence of magnesium and oxygen species was detected, which suggested that no contamination occurred during the synthesis processes.

### 2.3. Textural Characterization

**Figure 6:**
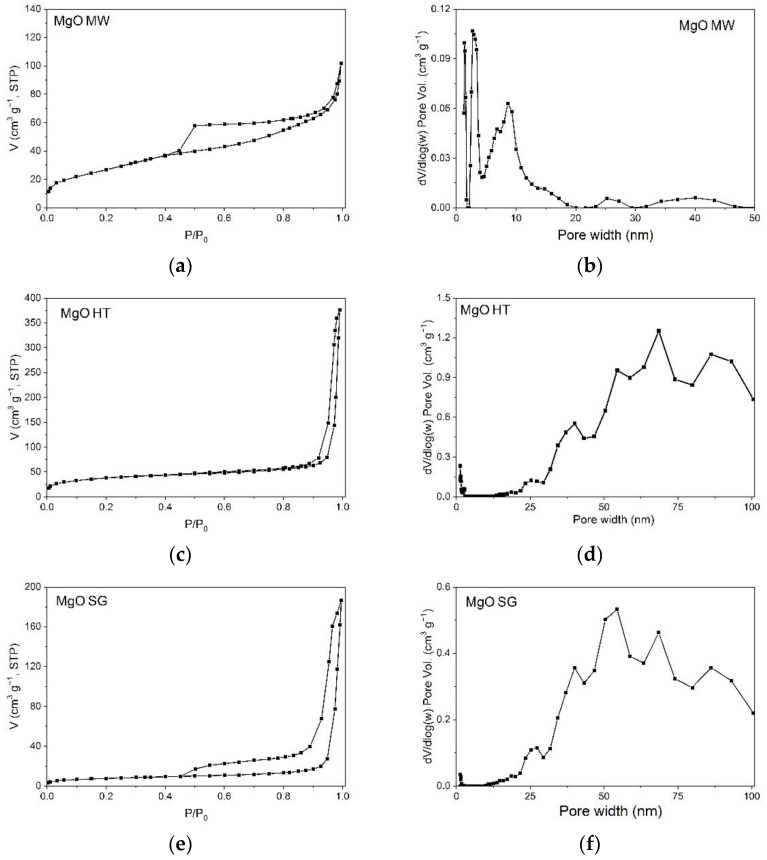
The nitrogen adsorption–desorption isotherms (**a**,**c**,**e**) and DFT pore size distributions (**b**,**d**,**f**) of the samples.

N_2_ adsorption–desorption measurements were carried out to investigate the textural features of the MgO samples. All samples exhibited type IV isotherms with H3-type hysteresis loops, as shown in Figure 6. According to IUPAC classification [48], this type of isotherm is a characteristic of mesoporous materials while H3 hysteresis loops appear in materials with flexible pores with a slit or plate-like morphology or those that form particle agglomerates. Unlike the MgO MW sample, whose pore distribution range had a relatively narrow interval (0–20 nm), for the MgO HT and MgO SG samples, the pore sizes were greater than 20 nm and even exceeded the range of mesopores (2–50 nm). As can be seen in Table 3, MgO HT had the highest BET specific surface area, followed by MgO MW and MgO SG. Regarding the total volume of the pores, the order was different, namely, MgO HT > MgO SG > MgO MW. These findings confirmed the presence of porous structures in the prepared MgO samples.

### 2.4. UV–Vis Spectroscopy Analysis

**Figure 7:**
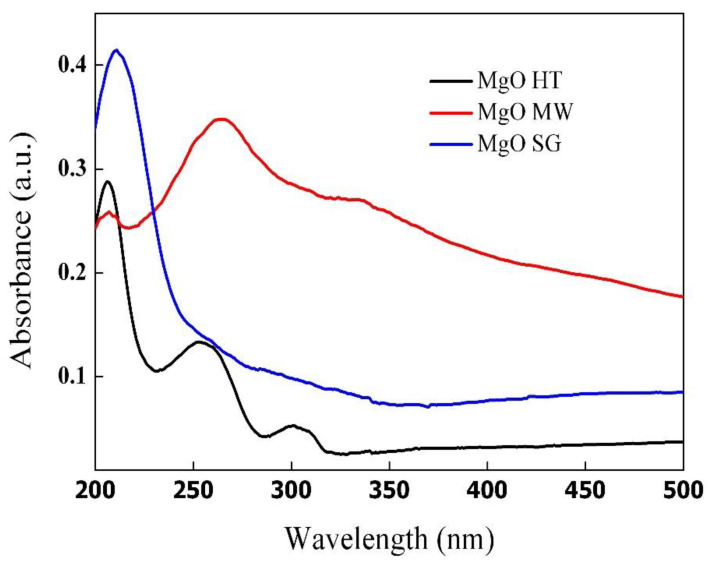
UV–Vis absorption spectra of the MgO powders prepared by the three different methods.

The optical properties of the MgO samples synthesized by the three different methods were evaluated using UV–Vis spectroscopy, and the recorded spectra are shown in Figure 7. For all three samples, the absorption bands with the maximum values located at ~206 nm were assigned to the excitation of five coordinated oxygen anions from the periclase structure [49]. Additional absorption bands located at higher wavelengths could be assigned to various F-type defects generated during synthesis. The most intense absorption bands were noticed in the case of the magnesium oxide prepared by the microwave method. Thus, the peak located at ~255 nm suggested the presence of F^+^ and F centers while the peaks recorded at wavelengths around 300 nm indicated the formation of some F_2_^2+^ centers [49]. The same absorption bands were also observed for the MgO sample obtained by the hydrothermal method, indicating the formation of several structural defects. It has been previously reported [50] that during hydrothermal treatment, the generation of water molecules between neighboring hydroxyl ions and their losses cause the occurrence of defects in the MgO’s structure. Furthermore, the formation of inter-crystallite channels and cracks and their contributions to higher specific surface areas than in the case of the sol–gel method (as was evident in the present study, see Table 3) was discussed [50].

The band gap energy for each sample was estimated by Tauc plots of the Kubelka–Munk function for direct transitions. The obtained results shown in Table 3 evidenced the effects of the synthesis methods on the bandgap energies of the MgO. Although magnesium oxide is a wide band-gap material (7.8 eV) [51], the synthesis methods proposed in the present work lowered the band gaps due to the formation of different defects that acted as intermediate levels available for the transfer of excited electrons [49]. The values of the obtained band gaps are shown in Table 3. Thus, the lowest value was obtained for magnesium oxide prepared by the microwave method (3.94 eV), indicating that this preparation method is the most suitable for obtaining magnesium oxide with improved photocatalytic properties.

### 2.5. Photoluminescence (PL) Studies

**Figure 8:**
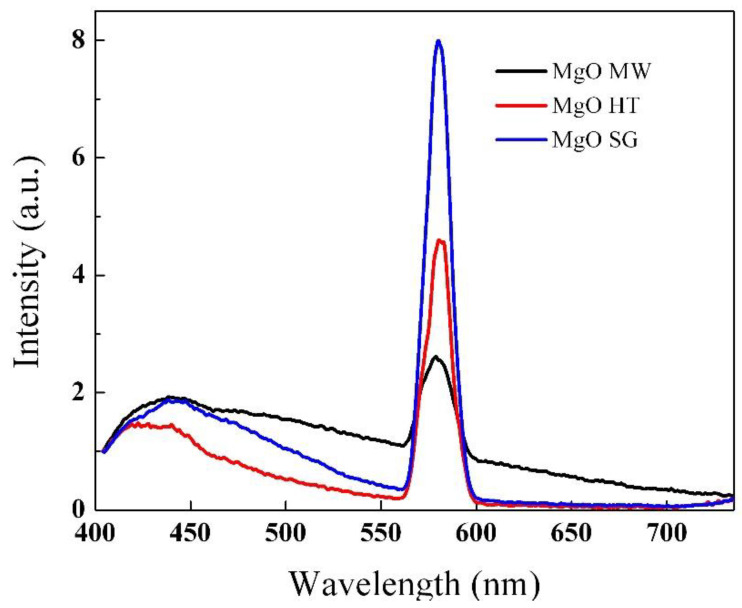
PL spectra of the MgO powders synthesized by the sol–gel (SG), microwave-assisted sol–gel (MW), and hydrothermal (HT) methods (λ_exc_ = 385 nm).

The photoluminescence (PL) spectra recorded for the samples prepared by the three different methods are shown in Figure 8. For all the samples, the PL results showed broad emission bands in the violet and blue regions of the visible spectrum (410–490 nm) and a more intense and sharper peak in the green region (580 nm). These were mainly related to the presence of several defects in the MgO structures, depending on the synthesis method. Thus, the first emission peak located at 416 nm was associated with F centers resulting from the removal of neutral O atoms in the magnesium oxide structure while the peak that appeared at 440 nm could be attributed to the dimmers of the F center, such as F_2_^2+^ [52,53]. The green emissions recorded for all the samples were mainly due to oxygen deficiencies in the structures of the synthesized magnesium oxides, as has been suggested in other studies [54,55].

### 2.6. Photocatalytic Activity

**Figure 9:**
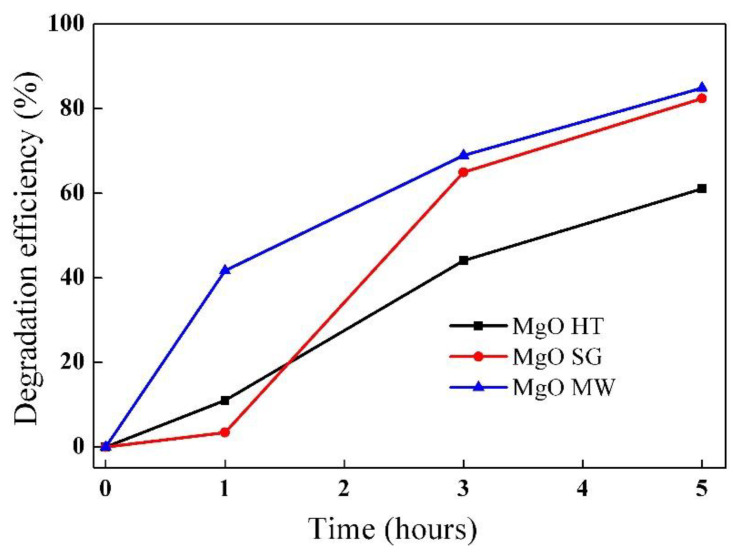
Photocatalytic performances of the MgO samples prepared by the three different methods (λ_irrad._ = 254 nm, 1 × 10^–5^ M MO, and 5 mg of photocatalyst).

The photocatalytic activities of magnesium oxide samples were investigated by carrying out the degradation of methyl orange (MO) dyes. The variations in MO degradation efficiencies over time in the presence of the MgO photocatalysts are shown in Figure 9. The highest photocatalytic activity was found for the MgO prepared by the microwave method due to the obtained properties, which were beneficial for photocatalytic applications. The large specific surface area, the low band gap energy, the presence of defects that contributed to delaying the recombination of the photogenerated charges, and the spherical morphology obtained by the microwave method were properties that had beneficial effects on its photocatalytic efficiency.

In the case of the other two samples obtained by the sol–gel and hydrothermal methods, although the efficiencies of methyl orange degradations were slightly lower, the photocatalytic results were comparable to those reported in the literature. A comparative analysis of these results is summarized in Table 4.

### 2.7. Identification of Reactive Species

**Figure 10:**
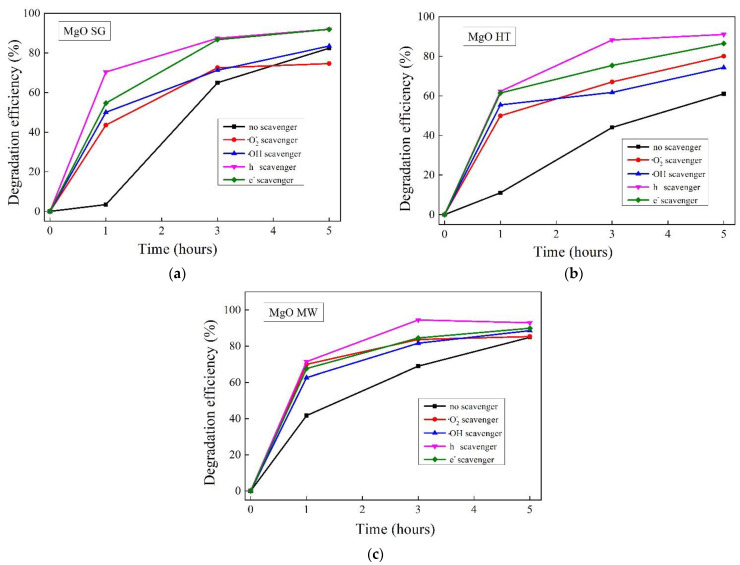
The effect of scavengers on the photocatalytic degradation of methyl orange by the MgO samples under UV light irradiation: (**a**) the sol–gel sample; (**b**) the hydrothermally prepared sample; and (**c**) the microwave-assisted sol–gel sample.

To understand the action pathway of each MgO sample, photocatalytic experiments were carried out in the presence of ·O_2_^−^, ·OH, and e^−^ and h^+^ scavengers. The results obtained are shown in Figure 10. A higher degradation efficiency was obtained in the case of adding the e^−^ and h^+^ scavengers. This behavior could be explained by the delay in the recombination of the e^−^/h^+^ pairs, which ensured a larger number of photo-generated species available for the degradation of the methyl orange. A slight increase in the photocatalytic efficiency was also obtained by adding ·OH and ·O_2_^−^ scavengers, which suggested an equal contribution of these reactive species to the degradation of the methyl orange. Therefore, when the superoxide anions were captured from the reaction medium, hydroxyl radicals (·OH), which are known as the most reactive oxygen species [58], became directly responsible for the degradation of the methyl orange molecules.

**Figure 11:**
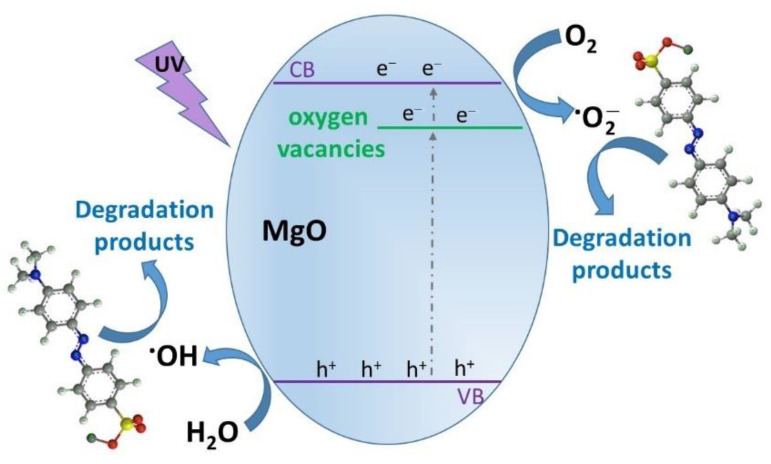
Schematic representation of the possible degradation mechanism of the methyl orange by the MgO samples.

Based on these results, it could be concluded that all the investigated active species had important contributions to the photocatalytic degradation of the methyl orange. Thus, by quenching a reactive species, its resultant lack was compensated by all the others in the system. A schematic representation of the possible degradation mechanism of the methyl orange by the MgO powders and the relevant reactive species can be found in Figure 11.

## 3. Conclusions

The present study aimed to correlate the structural and morphological properties of MgO powders generated by different synthesis methods with their photocatalytic activities. All the samples had porous, nanoscale, and microscale structures. The particles obtained by the sol–gel method had the lowest specific surface areas and the highest optical band gaps. The powders prepared by the microwave method had higher specific surface areas, narrow nanopore distributions, and the lowest band gaps. Additional UV-Vis absorption bands in the case of the MgO powders prepared by the microwave and hydrothermal methods indicated that defects were generated during the synthesis.

The best photocatalytic activity (~85% degradation efficiency) was obtained after 5 h of irradiation for the MgO synthesized by the microwave-assisted sol–gel method, for which the lowest band gap energy (3.94 eV) was obtained and spherical morphology. The microwave method induced the formation of several structural defects that prevented e^−^/h^+^ recombination. Thus, the photocatalytic process was improved by providing a high number of photogenerated charges responsible for the degradation of the MO.

## 4. Materials and Methods

### 4.1. Powder Preparation

The MgO powders were obtained by the sol–gel method using Mg(NO_3_)_2_ × 6H_2_O as a precursor in an ethanolic solution, H_2_O as a hydrolyzing agent, and NH_4_OH as the catalyst, according to the method presented in reference [59].

The hydrothermal synthesis started with the Mg(NO_3_)_2_ × 6H_2_O. The precipitating agent, NaOH, was dissolved in equal volume mixtures of water–ethanol, with the ratio of precursor/NaOH being 2/5, which was added to magnesium salt. The reaction system was kept under stirring until a white precipitate appeared, and then it was transferred to a hydrothermal cell heated at 130 °C, where it was kept for 14 h. After cooling at room temperature, the powder was separated by filtration, washed with distilled water, and dried, and then it thermally treated as mentioned in reference [59].

The microwave-assisted sol–gel method used Mg(CH_3_COO)_2_ × 4H_2_O in a 0.3 M aqueous solution and ammonia as a catalyst. The mixture was kept under microwaves for 10 min (2.45 GHz and 1 kW). The precipitated powder was treated as mentioned above, and then it was thermally treated as follows: 450 °C, 1 h plateau, and 1 °C/min.

### 4.2. Powders Characterization

The X-ray diffraction (XRD) patterns were recorded using a Rigaku Ultima IV X-ray diffractometer (Tokyo, Japan). The equipment was set in a parallel beam geometry with cross beam optics (CBO) and operated at 40 kV and 30 mA using CuKα radiation. The data were collected over the 2θ range at 10–85° at a scanning rate of 2 °/min. Rigaku’s PDXL v1.8 software, connected to the ICDD PDF-2 database, was used for phase identification.

SEM, TEM, and HRTEM coupled with SAED and EDX (energy dispersive X-ray spectroscopy) investigations were performed using a high-resolution FEI QUANTA INSPECT F scanning electron microscope (Thermo Fisher Scientific, Waltham, MA, USA) with a field emission gun and a TITAN THEMIS ultra-high resolution electron microscope (Thermo Fisher Scientific, Waltham, MA, USA). For the acquisition of the EDX spectra, the transmission electron microscope was operated in STEM (scanning transmission electron microscopy) mode at 300 kV.

The average particle sizes of the BST powder were estimated from the particle size distributions, which were determined using OriginPro 8.5 software (OriginLab, Northampton, MA, USA) by taking into account size measurements for ~30 particles, which were performed by means of the software of the electron microscope (Digital Micrograph 1.8.0) (Gatan, Sarasota, FL, USA).

The nitrogen physisorption isotherms were measured at −196 °C using an ASAP 2020 instrument from Micromeritics (Norcross, GA, USA). Before taking the measurements, the samples were outgassed under a vacuum at 250 °C for 4 h. The specific surface areas of the materials were assessed by the Brunauer–Emmet–Teller (BET) method, while the total pore volumes were calculated from the amounts adsorbed at relative pressures of 0.99. Pore size distributions were estimated using the density functional theory (DFT) method provided by the software of the ASAP 2020 instrument.

The morphologies of the MgO nanopowders were observed by field emission scanning electron microscopy (SEM) using a Quanta 3D FEG Dual Beam (Eindhoven, the Netherlands).

The UV–Visible absorption spectra of the MgO samples were recorded using a JASCO V570 spectrophotometer (Tokyo, Japan).

An FLSP 920 spectrofluorometer (Edinburgh Instruments, Livingston, UK) was used for recording the photoluminescence spectra (PL) of the magnesium oxide samples. The excitations at wavelengths of 385 nm were achieved with the help of an Xe lamp. The PL spectra were recorded at room temperature between 400 and 750 nm.

The photocatalytic reactions were carried out in a closed room at 30 °C.A total of 5 mg of MgO photocatalyst was added to a 10 mL solution of methyl orange dye (1 × 10^–5^ M). The mixture was firstly stirred in the dark for 30 min to allow the dye molecules to adsorb on the magnesium oxide surface. Then, irradiation at λ = 254 nm using a UV–VL-215c lamp was started, and it continued for 5 h. After 1, 3, and 5 h of irradiation, the photocatalyst was then separated from the suspension using a Millipore syringe filter (0.45 μm). The filtered solution was spectrophotometrically analyzed using the same JASCO V570 spectrophotometer to evaluate the dye degradation progress. The degradation efficiency was calculated as D_eff_ = (A_0_ − A_t_)/A_0_ × 100, where A_0_ is the absorbance of the initial MO solution and A_t_ is the absorbance at a particular interval of time.

To investigate the main active species responsible for the MO degradation, scavenger experiments were undertaken. In this regard, p-benzoquinone (p-BQ) was used as a quencher for the superoxide radicals (·O_2_^−^), ethanol was used for the hydroxyl radicals (·OH), silver nitrate was used for the electrons, and potassium iodide was used for the holes. The scavenger’s concentrations were 0.1 mM, and the reaction conditions were the same as in the photocatalytic experiments.

## Figures and Tables

**Table 1:** Literature survey of similar synthesis methods.

Methods and Parameters	Precursors/Catalysts/Solvents	Thermal Treatment	Crystalline Phases and Morphologies	Ref.
MW–360 W, 2 min, (on/off at 30 s)	Mg(CH_3_COO)_2_ × 4H_2_O benzylamine	550 °C for 5 h	MgO nanoparticles	[30]
MW–1 kW, 20 min (convection mode)	Mg(CH_3_COO)_2_ × 4H_2_O *A. paniculata* extract	400 °C for 2 h	MgO nanorods	[13]
MW–15 min	Magnesium nitrate, NaOH, H_2_O	400 °C for 4 h	MgO nanoparticles	[31]
MW–850 W, 15 min	Magnesium nitrate, urea, H_2_O	500 °C for 2 h	MgO nanosheets	[32]
HT–150 °C, 3 h	MgCl_2_ × 6H_2_O, 1-Propanol, Urea (or NaOH), 2,4,6-trinitrophenol, H_2_O	600 °C for 3 h	MgO nanorods (urea) and nanoparticles (NaOH)	[16]
HT–180 °C, 24 h	magnesium nitrate, NaOH, and H_2_O	400 °C for 2 h	MgO nanoparticles	[33]
HT–60 °C, 3–96 h	MgCl_2_, Na_2_CO_3_, and H_2_O	200 °C for 2 h	MgO spheroidal and flake (or) rectangular particles	[34]
HT–120 °C for 12 h	Mg(NO_3_)_2_ × 6H_2_O, CO(NH_2_)_2_), sodium dodecyl sulfate, and H_2_O	400 °C for 5 h	MgO nanosheets	[35]
HT–180 °C for 10 h	Mg(NO_3_)_2_ × 6H_2_O, H_2_O, and ethanol	500 °C for 3 h	MgO nanowires	[36]
HT–80–200 °C for 2 h	50 nm MgO particles and H_2_O	300 °C for 1 h, 450 °C for 1 h	MgO plate-like shape	[37]
HT–200 °C for 24 h	Mg(NO_3_)_2_ × 6H_2_O, urea, and H_2_O	500 °C for 5 h	MgO mesoporous	[38]
HT–150 °C for 24 h	Mg(NO_3_)_2_ × 6H_2_O, NaOH, and H_2_O	400 °C for 4 h	MgO flower-like shape	[31]
HT–130 °C for 6 h	I. (NH_4_)_2_CO_3_, Mg(NO_3_)_2_, and H_2_O II. nesquehonite, (NH_4_)_2_CO_3,_ and H_2_O	500 °C for 6 h	MgO random flakes, house-of-cards, spherical structures	[39]
HT–180 °C for 5 h	Mg(CH_3_COO)_2_ × 4H_2_O, urea, and H_2_O (pH 8) Mg(CH_3_COO)_2_ × 4H_2_O, urea, acetic acid, and H_2_O (pH 5–6) Mg(CH_3_COO)_2_ × 4H_2_O, urea, ammonia, and H_2_O (pH 9–10)	500 °C for 5 h	MgO mesoporous ball-like rhombohedrons (pH 5), smaller micro-rods (pH 9), and micro-rod-like (higher pH)	[40]
SG	0.1 mM Mg(NO_3_)_2_ × 6H_2_O, 0.1 M NaOH, and 100 mL H_2_O	400 °C for 4 h	MgO + Mg(OH)_2_	[31]
SG	Mg(NO_3_)_2_ × 6H_2_O and NaOH 1:2 M ratio of Mg^2+^ to OH^-^	400 °C for 5 h	MgO spherical nanoparticles	[14]
SG	8.96 wt. % Mg MeO in MeOH sol., PhMe, and MeOH hydrolysis ratio = 2 M vol. ratio PhMe: MeOH = 0.94	400 °C (vacuum)	MgO	[41]
modified thermal/SG	Mg(C_2_H_3_O_2_)_2_/Mg(NO_3_)_2_, NaOH/NH_4_OH, sodium dodecyl sulfate, and H_2_O	400–700 °C for 2 h	MgO + MgSO_4_ (traces), porous, agglomerated, and uniform semi-spherical flaky shape MgO	[15]
SG	Mg(CH_3_COO)_2_ × 4H_2_O C_2_H_2_O_4_ × 2H_2_O/C_4_H_6_O_6_, C_2_H_5_OH, and H_2_O (pH 5)	400 °C	MgO + MgC_2_O_4_	[42]
		500 °C	MgO nanocrystals	
SG	MgCl_2_ and C_6_H_8_O_7_ × H_2_O Mg^2+^: C_6_H_8_O_7_ = 1:3 T = 60 °C	500 °C for 2 h	MgO spherical particles	[43]
SG	Mg(NO_3_)_2_ × 2H_2_O, Pluronic P123, NH_4_OH (28%), and H_2_O pH 10 and T = 60 °C	600 °C for 2 h	MgO nanoparticles	[44]
SG	Mg(NO_3_)_2_ × 6H_2_O, NaOH, and H_2_O 30 min ultrasonic stirring	400 °C for 3 h	MgO nanosphere	[32]
SG	Mg(NO_3_)_2_ × 6H_2_O, NaOH, NH_4_OH, and H_2_O molar ratio Mg^2+^:OH^−^ = 1:2	500 °C for 4 h	MgO nanoparticles	[24]
SG	Mg(NO_3_)_2_ × 6H_2_O, NaOH, and H_2_O pH 12	500 and 800 °C for 4 h	MgO nanoparticles	[45]
SG	Mg(OCH_3_)_2_ and H_2_O 40 h at RT	500 and 600 °C for 4 h	MgO	[46]
SG	Mg(OCH_3_)_2_, C_2_H_5_OH, HCl, NH_4_OH, and H_2_O pH 9 and 40 h reflux	400, 600 and 800 °C for 2 h	MgO particles	[47]

**Table 2:** Phase compositions, lattice parameters, crystallite sizes, and quantitative ratios.

Sample	Phase(s)	Lattice Parameters (Å)	Crystallite Size, *D*_XRD_ (nm)	Quantitative Ratio (%)
MW	Periclase, MgO	4.22569(5)	10	100
HT	Periclase, MgO	4.23557(17)	6	88.5
	Brucite, Mg(OH)_2_	-	-	7.7
	Hydromagnesite, Mg_5_(CO_3_)_4_(OH)_2_ × 4H_2_O	-	-	3.8
SG	Periclase, MgO	4.21986(11)	15	87.7
	Brucite, Mg(OH)_2_	-	-	3.3
	Hydromagnesite, Mg_5_(CO_3_)_4_(OH)_2_ × 4H_2_O	-	-	9

**Table 3:** The BET specific surface areas (S_BET_), total pore volumes (V_total_), and band gaps of the samples.

Sample	S_BET_ (m^2^g^−1^)	V_total_ (cm^3^g^−1^)	Bandgap Energy (eV)
MgO MW	101.1	0.157	3.94
MgO HT	132.1	0.562	4.48
MgO SG	26.3	0.289	5.32

**Table 4:** A comparison of the photocatalytic results under UV light irradiation of MgO samples synthesized by different methods.

Method	Phase	Band Gap (eV)	Degradation Efficiency (%)	Ref.
Thermal decomposition of Mg(OH)_2_ by the wet chemical method	Polycrystalline MgO with a cubic structure	5.54	50% of methyl orange (10 mg/L)	[56]
Green synthesis	Cubic MgO structure	4.17	81% of methylene blue (20 ppm)	[57]
Reflux condensation approach	Cubic MgO structure	5.63	92% of methyl orange 95% of methylene blue	[1]
		5.67	96% of methyl orange 99% of methylene blue	
Combustion method	Polycrystalline cubic structure of MgO nanoparticles	-	75% of methylene blue	[4]
Sol–gel method	Periclase, MgO brucite, Mg(OH)_2_ hydromagnesite, and Mg_5_(CO_3_)_4_(OH)_2_ × 4H_2_O	5.32	82% of methyl orange (1 × 10^−5^ M)	Present work
Hydrothermal method	Periclase, MgO brucite, Mg(OH)_2_ hydromagnesite, and Mg_5_(CO_3_)_4_(OH)_2_ × 4H_2_O	4.48	61% of methyl orange (1 × 10^−5^ M)	
Microwave-assisted sol–gel method	Periclase, MgO	3.94	85% of methyl orange (1 × 10^−5^ M)	

## Data Availability

All data are available upon reasonable request from the authors.
